# Inequalities in patients’ experiences with cancer care: the role of economic and health literacy determinants

**DOI:** 10.1186/s12913-024-11174-x

**Published:** 2024-06-14

**Authors:** Vladimir Jolidon, Manuela Eicher, Isabelle Peytremann-Bridevaux, Chantal Arditi

**Affiliations:** 1 Unisanté, University Center for Primary Care and Public Health, Department of Epidemiology and Health Systems, University of Lausanne, CH-1011 Lausanne, Switzerland; 2 Institute of Higher Education and Research in Healthcare (IUFRS), Faculty of Biology and Medicine, University of Lausanne, Lausanne, Switzerland; 3 Department of Oncology, Lausanne University Hospital (CHUV), Lausanne, Switzerland

**Keywords:** Cancer, Patient experience, Patient survey, Patient satisfaction, Health literacy, Socioeconomic status, Access, Information, Coordination, Communication, Continuity, Emotions, Needs, Quality of care

## Abstract

**Background:**

Patients with fewer socioeconomic and health literacy resources are disadvantaged in their access and use of healthcare, which may give rise to worse experiences with care and thus inequalities in patient experiences. However, only a limited number of studies have examined how socioeconomic and health literacy factors shape inequalities in patients’ experiences with cancer care.

**Objective:**

To examine whether patients’ experiences with cancer care differ according to their economic status and health literacy.

**Methods:**

Secondary analysis of data on 2789 adult patients diagnosed with cancer from the Swiss Cancer Patient Experiences-2 (SCAPE-2) study, a cross-sectional survey conducted in eight hospitals across Switzerland from September 2021 to February 2022. Regression analysis was applied to examine the independent effect of patients’ economic status and health literacy on various outcomes of experiences with cancer care, covering eight different dimensions of patient-centred care, controlling for confounding factors.

**Results:**

Adjusted regression analysis showed that patients with lower economic status reported significantly worse experiences with cancer care in 12 out of 29 specific care experiences, especially in the dimensions of ‘respect for patients’ preferences’ and ‘physical comfort’ where all items of experiences were associated with economic status. Additionally, lower health literacy was associated with worse patient experiences in 23 specific care experiences. All items in the dimensions of ‘respect for patients’ preferences’, ‘physical comfort’ and ‘emotional support’ were associated with health literacy.

**Discussion:**

This study revealed significant inequalities in experiences with cancer care shaped by the economic status and health literacy of patients across different dimensions of patient-centred care. It is essential to address the needs of more disadvantaged patients who face obstacles in their access and use of the healthcare system, not only to mitigate inequalities in cancer care but also to avoid inequalities in health outcomes.

## Introduction

Patient experience of care is acknowledged as a key element of the quality of healthcare and relates to safety of care and clinical effectiveness [1, 2]. Hence, it is included in performance frameworks evaluating the quality of healthcare. Patient experience is typically assessed with patient-reported experience measures (PREMs), which collect information on the care received by patients, their interactions with health professionals and the outcomes of those interactions [3–7]. PREMs were developed to capture the experiences of patients with various care events and to provide specific and practical information that may be acted upon to improve patient care. They differ from patient satisfaction measures, which cover a limited area of patient experience with care and provide a narrower perspective compared to the multiple facets of patient experiences [8, 9]. Importantly, PREMs have been regarded as an accurate indicator of patient-centredness in healthcare. Patient-centredness is a dimension of quality of care that is defined as care provision responsive to and respectful of patient needs, preferences, and values [10, 11].

Most commonly, overall levels of patient experience with care (i.e. the proportion of patients reporting a positive experience with care) are reported to assess healthcare services, with little examination of possible variations between subgroups of patients. That is, patients’ experiences may be shaped by their sociodemographic profiles and access to resources, which can result in inequalities in care experiences when more disadvantaged patients consistently report poorer experiences. The perception and experience of vulnerable patients with care are critically important yet poorly understood dimension of healthcare delivery. Indeed, limited attention has been paid to how individuals’ socioeconomic status may give rise to inequalities in care experiences, although health equity is a central goal for healthcare improvement in the “quintuple aim” framework [12]. This is particularly important since patient experiences may affect healthcare access and health outcomes [13, 14], and inequalities in patient experience may thus exacerbate disparities in health and quality of life.

Previous research has shown that factors such as age, gender, ethnicity, cancer prognosis and health status may influence patients’ experiences with care [15–17]. Nevertheless, there has been a relative lack of attention given to the effect of socioeconomic status and particularly health literacy on patient experiences, despite recent studies suggesting that these factors may affect care experiences [18–20]. In sum, it is essential to investigate social inequalities in PREMs as a key element of quality of care. Results of such research can contribute to a better understanding of specific groups of patients who require further attention, in order to address their needs and enhance their care experience, and in turn, their health outcomes.

### Socioeconomic status and patient experiences in cancer care

Past studies have shown socioeconomic inequalities in patient satisfaction and care experiences. Lower income patients and those from deprived neighbourhoods tend to report lower satisfaction and worse experiences with healthcare services compared to more affluent patients [21–28]. In the field of cancer care, previous studies have revealed sociodemographic differences in patient care experiences, such as age, ethnicity and gender differences [15, 16, 29–31]. However, these studies were mostly conducted in the United Kingdom and the United States of America [17] and research on socioeconomic inequalities in cancer patients’ experiences remains limited.

Inequalities in patient experience may be explained from the standpoint of the fundamental cause theory [32]. According to this framework, social conditions act as fundamental causes of individual health since the resources available to individuals, such as knowledge, power, money and social networks, determine their ability to avoid diseases and protect their health [32]. While this theory does not directly address healthcare use and experience, its authors asserted that fundamental social conditions “directly shape individual health behaviours by influencing whether people know about, have access to, can afford and are motivated to engage in health-enhancing behaviours” [32]. Hence, socioeconomically disadvantaged patients may face more barriers in healthcare use compared to their more advantaged counterparts, and may experience unmet healthcare needs stemming from the (direct and indirect) costs of healthcare [33–35]. In sum, a vulnerable condition defined by lower socioeconomic status and limited financial resources may negatively affect patients’ experiences of healthcare services.

### The role of health literacy

In addition to socioeconomic resources, patient’s cultural skills and competencies may influence their experience with care. Thus, the fundamental cause theory is complemented by the “cultural health capital” framework, which stresses that patients’ encounters with healthcare services are defined by their cultural resources [36]. That is, patients who have an “enterprising and proactive disposition, a fluency in biomedical concepts and language, bureaucratic know-how, and an interactional agility with authoritative experts” [36] are better equipped to navigate the complexities of the healthcare system. Such individual characteristics also facilitate their interactions and exchange of informations with healthcare professionals. Consequently, inequalities in care experiences may stem from differences in patients’ cultural abilities, and the dynamics of unequal treatment that these engender in the healthcare context [37].

In this framework, health literacy is a key characteristic that determines patients’ ability to communicate and function in the healthcare environment. It is defined as “the degree to which individuals have the capacity to obtain, process, and understand basic health information and services needed to make appropriate health decisions” [38]. Individuals with lower health literacy may experience difficulty obtaining, understanding, and retaining health information, which is associated with the under-utilisation of (preventive) healthcare services [18, 39], more frequent hospitalisations and emergency care utilisation [40, 41], worse health outcomes and increased mortality [42, 43]. These associations were also found among patients with cancer [44–47]. Moreover, lower health literacy was shown to relate to the inefficiency of consultations and dissatisfaction with healthcare [18, 19, 48, 49], as well as lower health-related quality of life among patients with cancer [50, 51].

Although health literacy has been studied in different areas of cancer care [52], no study has examined health literacy-related inequalities across a range of PREMs encompassing diverse dimensions of patient care experiences. Thus, it is important to elucidate the association of health literacy with various care experiences, especially since research has evidenced its independent association with various health outcomes, health-related behaviours, and health service use [53]. While health literacy levels may indeed relate to people’s socioeconomic conditions, this relationship can be more complex and influenced by further factors, such as family and social support networks. Hence, health literacy should not solely be considered as a mediator, and its potential direct effect on care experiences warrant analysis.

Finally, health literacy directly relates to a patient-centred care approach that places patients’ perspectives, values, needs and preferences as the starting point and the importance of communication and information [10, 19]. Indeed, for patients to be empowered to understand health information and make decisions about their own care, they should have the necessary education, information, and support [19, 53, 54]. However, if health literacy is unequally distributed, as well as support for patients, inequalities in patients’ experiences and health outcomes may persist.

In that context, the present study aims to investigate inequalities in cancer care experiences shaped by patients’ economic and health literacy resources. This is particularly relevant since a limited number of studies have focused on such inequalities in patients’ experiences, especially in the field of cancer care and in countries other than the UK and USA.

## Methods

### Study design, population, and data

The present study is a secondary analysis of data from the second wave of the Swiss Cancer Patient Experiences-2 (SCAPE-2) study. This cross-sectional and multicentre survey collected data from patients diagnosed with cancer in eight Swiss hospitals between September 2021 and February 2022. The survey inclusion criteria were adult patients (18 years of age or older) who had a cancer diagnosis, at least one cancer-related hospitalisation or outpatient visit at one of the recruiting hospitals between January 1 and June 30, 2021, and a home address in Switzerland. The patient selection procedure was carried out by local hospital teams, manually reviewing patient lists or using electronic databases. The hospitals were recruited by sending invitation letters to the oncology departments of 13 hospitals, including all five university hospitals as well as those with large oncology departments. Eight hospitals agreed to participate in the study, achieving a broad representation of Swiss hospitals. Notably, four were located in the French-speaking region and four in the German-speaking region, each situated in a different canton. Among them were three university hospitals and five cantonal (regional) hospitals, with two located in major Swiss cities (Geneva and Zurich), four in medium-sized cities (Lausanne, Lucerne, Zug, and Fribourg), and two in smaller cities (Chur and Sion) situated in more rural cantons. The hospitals varied in size, with four having between 7,000 and 12,000 employees and four having between 1,000 and 6,000 employees.

The data was collected using the SCAPE-2 questionnaire, a self-administered questionnaire based on the NHS Cancer Patients Experience Survey [55]. The questionnaire, translated and culturally adapted from the original version developed in the United Kingdom, contained 130 questions in total, including patient experience, health, and socio-demographic questions. Respondents had the option to fill it out on paper and return it by post, or to complete it online. The questionnaire was sent to patients’ homes by each hospital in September 2021, and reminders were sent to non-respondents in November 2021. Patients who returned the questionnaire by the end of February 2022 were included in the study. Out of 6873 patients who were invited to take part in the survey, 3220 completed it (crude participation rate 46.8%).

### Outcome variables

Of the 71 patient experience questions, we selected 29 questions pertaining to experiences regarding cancer care in general that applied to all patients as outcomes in this study. These questions covered the cancer care pathway, from pre-diagnostic care to home care, and assessed the eight dimensions of patient-centred care. Building upon the work of Gerteis and colleagues [56], the Picker Institute developed these dimensions, capturing the essential components of care quality [57, 58]:

- Access to care: waiting time before seeing a specialist (before_wait).
- Information and education: understood diagnostic explanations (dx_explanation), received written diagnostic information (dx_information), treatment options were explained (ttt_opt), treatment side effects explained in understandable way (ttt_sidefx_expl), told about long-term side effects (ttt_sidefx_future), received information on impact of cancer on daily activities (info_impact), received information on support groups (info_support_gp), received information on getting financial help (info_support_fin), receiving a care plan (careplan).
- Coordination and integration of care: professionals working well together (collaboration), test results or medical records available (test_avail), receiving clear and consistent information (info_consist), Test not repeated (test_repet).
- Continuity and transition: support and equipment available at home (home_support), care from health or social services during (home_service_during) or after (home_serv_after) treatment, GP receiving information (gp_info).
- Involvement of family and friends: told that could be accompanied (dx_accompanied), family involved in treatment decisions (ttt_dec_fam), family was given information for care at home (home_info_fam).
- Respect for patients’ preferences: involved in treatment decisions (ttt_involve), situation and habits taken into account (ttt_dec_hab).
- Physical comfort: information/support to deal with symptoms (ttt_support_symp), information/support to deal with long-term effects (support_ltsidefx).
- Emotional support: told in a sensitive manner (dx_tactful), offered to see health professional to discuss worries (support_worries), GP support (gp_support), received information on support to handle emotions (info_support_emo).

Most experience questions had 5-point Likert-type scale response options to measure positive experiences (i.e. ‘yes, completely’; ‘yes, to some extent’; ‘no’; ‘not applicable’; and ‘don’t know/can’t remember’). We computed binary variables for each question to capture patients’ positive experiences (response ‘yes, absolutely’) versus non-positive experiences (responses ‘yes, to some extent’ and ‘no’), while neutral (‘don’t know/can’t remember’) and not applicable answers were excluded, following the methodology of the NHS Cancer Patients Experience Survey [55] and previous similar studies [16, 59]. The dichotomisation of outcome variables facilitates clearer presentation, interpretation, and comparison across the numerous outcome measures assessed.

### Independent variables

The first independent variable of interest, ‘economic status’, was computed using the following three questions: ‘In the past 12 months, have you had difficulty paying for your bills (taxes, insurances, telephone, electricity, credit card, etc.)?’, ‘In the past 12 months, have you skipped any medical care because of the cost?’ and ‘Have you or your family had to make financial sacrifices because of treatment or the long term effects of cancer?’. These variables were dichotomised (yes = 1, no = 0) and summed into a score from 0 to 3. A higher score entailed more economic hardship and thus a lower economic status, that is, patients who answered that they faced economic hardships in the three questions obtained a score of 3. The second independent variable of interest, ‘health literacy’, was obtained from a question assessing patients’ difficulty in understanding written medical information, which has shown to have good sensitivity and specificity in capturing individuals’ health literacy limitations [60]: ‘When you receive written information concerning a medical treatment or your health, do you have problems understanding it?’ to which respondents could respond ‘always’, ‘often’, ‘sometimes’, ‘occasionally’, or ‘never’. Answers were recoded into a binary variable with the categories ‘low health literacy’ (always, often) and ‘high health literacy’ (sometimes, occasionally, never). Finally, other independent variables of our study were patients’ age (continuous variable), sex (woman, man) and self-rated health (5-point scale: 0 ‘bad’, 25 ‘average’, 50 ‘good’, 75 ‘very good’, 100 ‘excellent’).

### Statistical analyses

After the exclusion of patients with missing information for the independent variables (*n* = 431; 13.4%), the study sample contained 2789 individuals. As the 29 outcomes, analysed in separate models, had different amounts of missing information, the final sample size for each of these outcomes is reported in Table 1. First, we performed univariate analysis to describe the independent (patient sociodemographic and health characteristics) and dependent (patient care experiences) variables of the study. Then, we conducted multiple logistic regression analyses to examine the association between economic status and health literacy and 29 outcomes of patient experiences with care, in separate models. Model 1 only included the independent variable of interest – economic status or health literacy - and hospital fixed effects (one dummy variable for each hospital except one) to control for between-hospital heterogeneity. Model 2 was adjusted for age, sex, self-rated health, health literacy, economic status and hospital fixed effects. Odds ratios (ORs) with 95% confidence intervals were graphically presented, for ease of interpretation of the results.

**Table 1:** Percentage of positive experience in cancer care across eight patient-centred care dimensions

	*N*	Positive experiences *n* (%)
*Access to care*		
Waiting time before seeing a specialist was adequate (before_wait)	2010	1651 (82.1)
*Information & education*		
Understood diagnostic explanations (dx_explanation)	2733	2022 (74.0)
Received written diagnostic information (dx_information)	1824	1035 (56.7)
Treatment options were explained (ttt_opt)	2407	2004 (83.3)
Treatment side effects explained in understandable way (ttt_sidefx_expl)	2651	1975 (74.5)
Told about long-term side effects (ttt_sidefx_future)	2471	1354 (54.8)
Received information on impact of cancer on daily activities (info_impact)	1903	1612 (84.7)
Received information on support groups (info_support_gp)	1356	1121 (82.7)
Received information on getting financial help (info_support_fin)	1095	597 (54.5)
Receiving a care plan (careplan)	2190	899 (41.1)
*Coordination & integration*		
Professionals working well together (collaboration)	2688	2304 (85.7)
Test results or medical records available (test_avail)	2666	2268 (85.1)
Receiving clear and consistent information (info_consist)	2675	2316 (86.6)
Test not repeated (test_repet)	2704	2386 (88.2)
*Continuity & transition*		
Support and equipment available at home (home_support)	1225	800 (65.3)
Care from health/social services during treatment (home_service_during)	1089	740 (68.0)
Care from health/social services after treatment (home_serv_after)	658	374 (56.8)
GP receiving information (gp_info)	2313	2141 (92.6)
*Involvement of family and friends*		
Told that could be accompanied (dx_accompanied)	2424	1499 (61.8)
Family involved in treatment decisions (ttt_dec_fam)	2319	1715 (74.0)
Family was given information for care at home (home_info_fam)	1242	771 (62.1)
*Respect for patients’ preferences*		
Involved in treatment decisions (ttt_involve)	2711	2004 (73.9)
Situation and habits taken into account (ttt_dec_hab)	1955	1383 (70.7)
*Physical comfort*		
Information/support to deal with symptoms (ttt_support_symp)	2250	1587 (70.5)
Information/support to deal with long-term effects (support_ltsidefx)	1943	1086 (55.9)
*Emotional support*		
Told in a sensitive manner (dx_tactful)	2705	2168 (80.1)
Offered to see health professional to discuss worries (support_worries)	1506	1255 (83.3)
GP support (gp_support)	2141	1672 (78.1)
Received information on support to handle emotions (info_support_emo)	1332	1079 (81.0)

In sensitivity analyses, we replicated Models 2 using economic status as a categorical variable (instead of a continuous score variable) with four categories from 0 (no economic hardship reported by the patient) to 3 (economic hardship were reported in all three questions), zero (0) being the reference category. We also conducted sensitivity analyses for multiple testing using Benjamini and Hochberg False Discovery Rate [61] to correct p-values, which is a method to ensure that we do not over-interpret a few significant results that may occur (by chance) when multiple tests are performed. As all statistically significant associations remained significant after applying the multiple testing correction, we did not present the corrected p-values in our figures (available on request). An additional sensitivity analysis was performed on the adjusted models (Models 2) where the type of cancer was added as a control variable to ensure that it did not confound the association between health literacy/economic status and patients’ care experiences.

Multicollinearity between independent variables was tested with variance inflation factors and no potential issues of collinearity were detected. All analyses were performed using Stata BE 17.0.

### Patient involvement

Patient involvement in research ensures that the research is relevant for patients and the larger public, that the research objectives are acceptable and feasible, and that the research and its documents are appropriate and comprehensive [62]. Two patients, trained and experienced in patient involvement in research, contributed to the development and implementation of the SCAPE-2 survey. They were recruited via the patient advisory board of the Swiss clinical cancer research organisation and involved in all steps of the research process. That is, they contributed to developing and pre-testing the questionnaire, preparing the materials sent to patients along with the questionnaire, replying to patients’ queries during the recruitment period, analysing questionnaires’ free-text comments, writing lay summaries of results shared with participating patients, and disseminating results on social media and to the scientific community.

## Results

Table 2 presents a summary of respondents’ characteristics. On average, they were 64 years old and 51% of them were women. The mean self-reported health was 54 on a scale from 0 to 100. Around one-fourth of the respondents reported a low level of health literacy (problems understanding written health information), and the mean score for economic status was 0.5 on a scale from 0 to 3.

**Table 2:** Patients’ sociodemographic and health characteristics (*N* = 2789)

	*N* (%) or Mean (SD)
Sex	
Women	1418 (50.8)
Men	1371 (49.2)
Age (Min = 18; Max = 96)	
Mean (SD)	63.9 (13.4)
Self-rated health (Min = 0; Max = 100)	
Mean (SD)	54.4 (20.4)
Health literacy	
Low	684 (24.5)
High	2105 (75.5)
Economic status (Min = 0; Max = 3)	
Mean (SD)	0.5 (0.8)

Patients’ experiences with cancer care across eight dimensions of patient-centred care are presented in Table 1. In the dimensions of ‘access to care’ and ‘coordination and integration’, more than 80% of patients reported positive experiences in all items. More than 80% of patients also reported positive experiences in other dimensions for specific items such as ‘treatment options were explained’, ‘received information on impact of cancer on daily activities’, ‘received information on support groups’, ‘GP receiving information’, ‘told in a sensitive manner’, ‘offered to see health professional to discuss worries’ and ‘received information on support to handle emotions’. In the dimensions of ‘involvement of family and friends’ and ‘respect for patients’ preferences’, about 60–75% of the patients reported positive experiences with cancer care. Finally, in the dimension ‘information and education’, 4 out of 9 items had proportions of positive experience lower than 60%, and this was also the case for specific items such as ‘care from health/social services after treatment’ and ‘information/support to deal with long-term effects’, falling under the dimensions ‘continuity and transition’ and ‘physical comfort’, respectively.

**Fig. 1:**
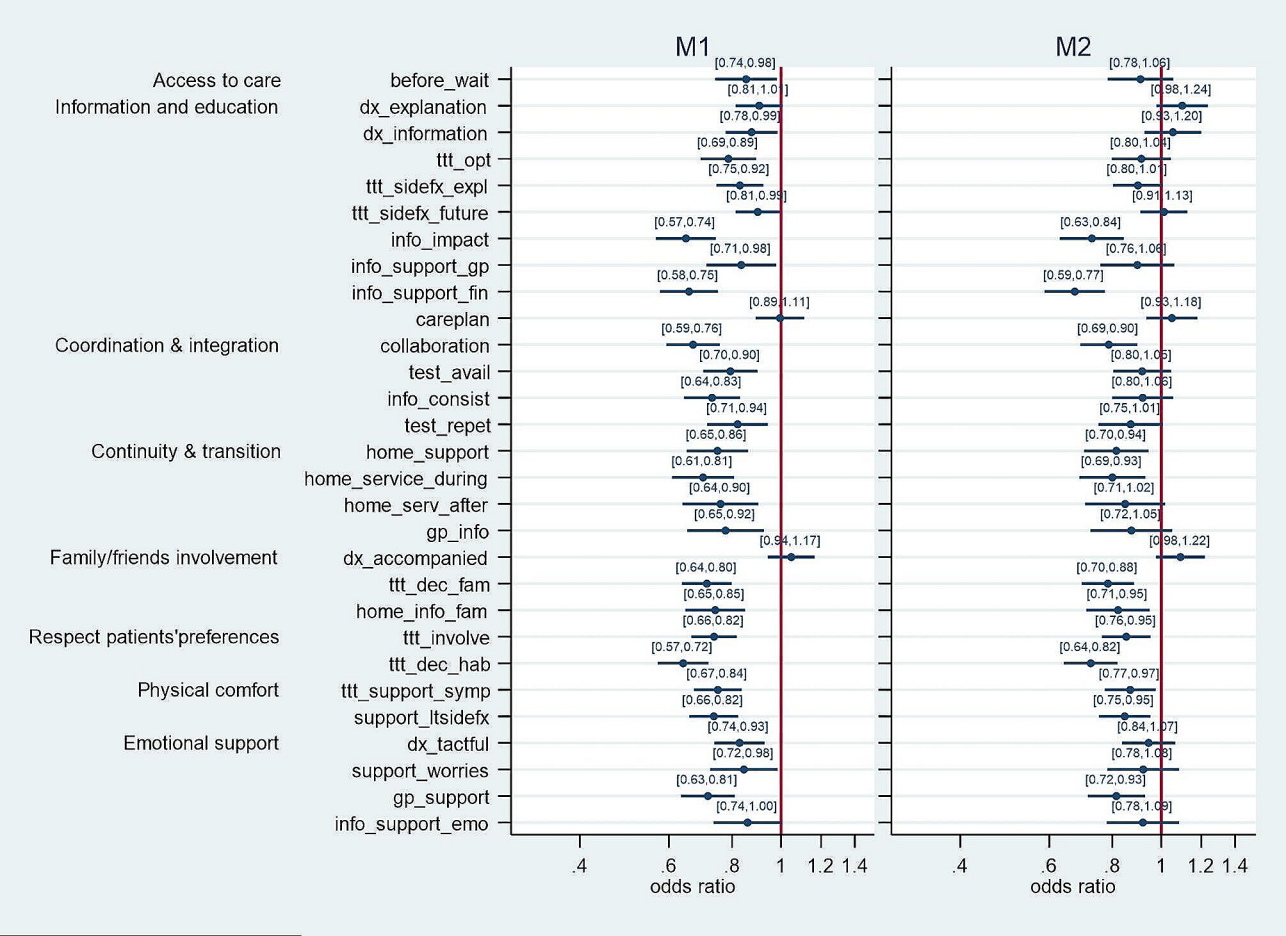
Effect of economic status on patients’ experiences with cancer care, odds ratio and 95% confidence intervals, unadjusted (M1) and adjusted models (M2). Note: M1 only included the independent variable ‘economic status’ and hospital fixed effects, and M2 added sex, age, self-rated health, and health literacy as control variables

The effect of patients’ economic status on their experiences with cancer care is shown in Fig. 1. In the unadjusted models (M1), a lower economic status was significantly associated with a lower probability of reporting positive care experiences in 25 out of 29 specific cancer care experiences. In the adjusted models (M2), economic status remained associated with 12 out of 29 cancer care experiences. Specifically, all items in the dimensions ‘respect for patients’ preferences’ (ttt_involve, ttt_dec_hab) and ‘physical comfort’ (ttt_support_sympt, support_ltsidefx) were associated with economic status in Models 2. The other dimensions had either one or two items associated with economic status (info_impact, info_support_fin, collaboration, home_support, home_service_during, ttt_dec_fam, home_info_fam, gp_support) except for ‘access to care’ (before_wait), in which the single item ‘waiting time before seeing a specialist was adequate’ was not found to be associated.

**Fig. 2:**
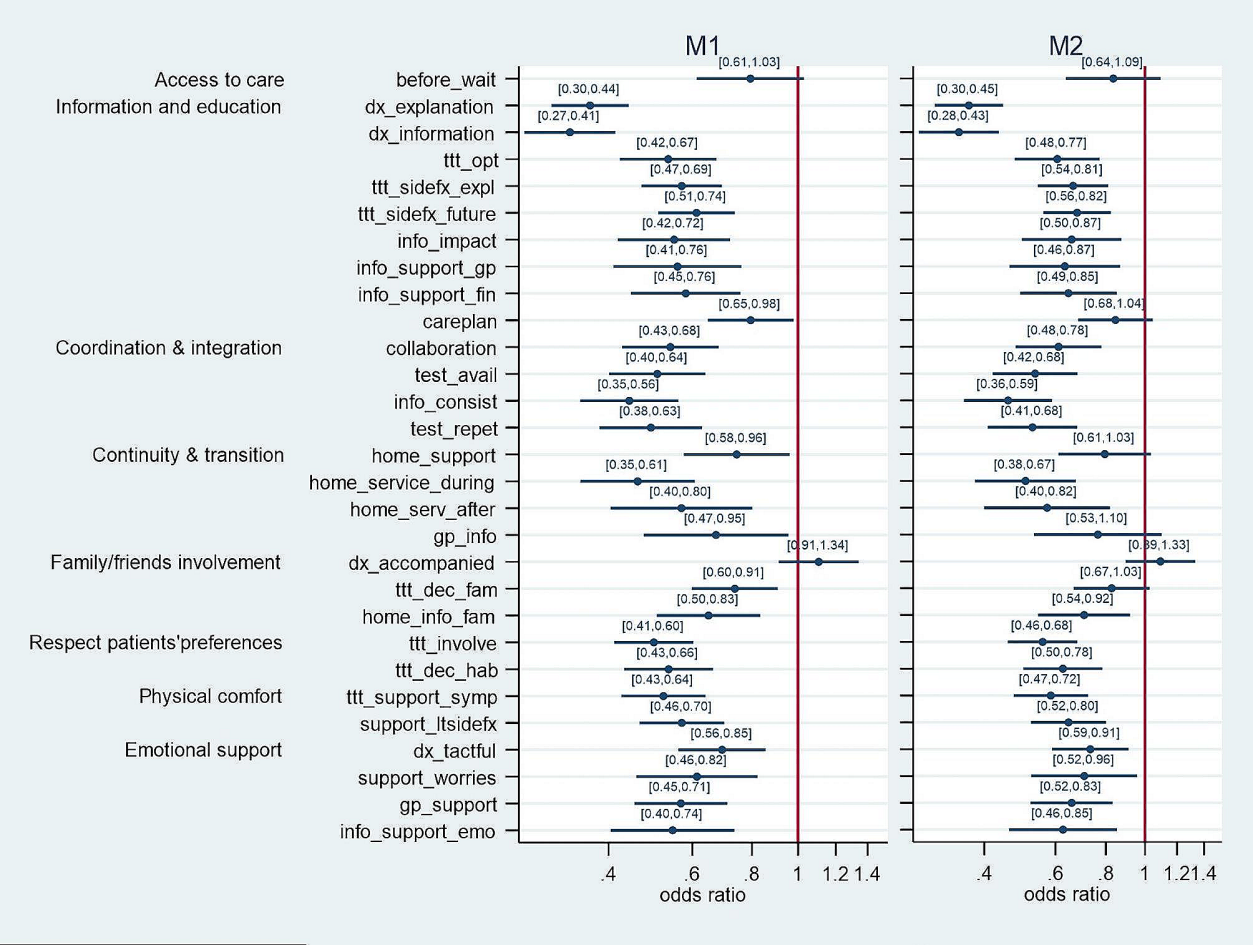
Effect of health literacy on patients’ experiences with cancer care, odds ratio and 95% confidence intervals, unadjusted (M1) and adjusted models (M2). Note: M1 only included the independent variable ‘health literacy’ and hospital fixed effects, and M2 added sex, age, self-rated health, and economic status as control variables

Concerning the effect of patients’ health literacy on their experiences with cancer care (Fig. 2), unadjusted models (M1) showed that patients’ lower health literacy was significantly associated with a lower probability of reporting positive care experiences in 27 out of 29 specific cancer care experiences. In the adjusted models (M2), health literacy remained associated with 23 out of 29 specific cancer care experiences. In particular, all items in the dimensions ‘respect for patients’ preferences’ (ttt_involve, ttt_dec_hab), ‘physical comfort’ (ttt_support_sympt, support_ltsidefx), ‘coordination and integration’ (collaboration, test_avail, info_consist, test_repet) and ‘emotional support’ (dx_tactful, support_worries, gp_support, info_support_emo) were associated with health literacy in Models 2, as well as all items except one in the ‘information and education’ (dx_explanation, dx_information, ttt_opt, ttt_sidefx_expl, ttt_sidefx_future, info_impact, info_support_gp, info_support_fin) dimensions. The dimensions of ‘continuity and transition’ and ‘involvement of family and friends’ had two (home_service_during, home_serv_after) and one item (home_info_fam) associated with health literacy, respectively, and the single item in ‘access to care’ (before_wait) was not found to be associated with health literacy.

When comparing the adjusted models (Model 2) of economic status and health literacy in Figs. 1 and 2, we observe that these two predictors were associated with ten common cancer care experiences (info_impact, info_support_fin, collaboration, home_service_during, home_info_fam, ttt_involve, ttt_dec_hab, ttt_support_symp, support_ltsidefx, gp_support). Health literacy alone was significantly associated with 13 additional care experiences (dx_explanation, dx_information, ttt_opt, ttt_sidefx_expl, ttt_sidefx_future, info_support_gp, test_avail, info_consist, test_repet, home_serv_after, dx_tactful, support_worries, info_support_emo).

In sensitivity analyses, Model 2 was replicated using economic status as a categorical variable. Out of the 12 cancer care experiences that were found to be significantly associated with economic status as a continuous (score) variable, six were associated with all three categories of economic status, showing clear gradients throughout the ORs of the categories (info_impact, info_support_fin, ttt_involve, ttt_dec_hab, support_ltsidefx, gp_support). The remaining six care experiences (home_info_fam, home_support, home_service_during, collaboration, ttt_support_symp, ttt_dec_fam) were associated with only one or two categories and showed relatively clear gradients throughout the categories (tables available upon request to the authors). In the sensitivity analysis where type of cancer was added to Models 2 (adjusted models), the results remained robust, with no substantial changes in the ORs of health literacy and economic status variables, and their statistical significance (results available upon request to the authors). Given that type of cancer did not affect the results, it was not included in the final models to avoid overfitting.

## Discussion

This study showed substantial inequalities in patient experiences, related to economic and health literacy factors, among patients with cancer treated in eight hospitals located in Switzerland. In adjusted models, lower economic status and lower health literacy were associated with worse care experiences in 12 and 23 out of 29 specific care experiences, respectively. Economic status affected all experience outcomes within the patient-centred care dimensions related to ‘respect for patients’ preferences’ and ‘physical comfort’, and health literacy affected all items in the dimensions of ‘respect for patients’ preferences’, ‘physical comfort’ and ‘emotional support’. Importantly, the effect of economic status and health literacy on the different experience outcomes remained significant after applying the multiple testing correction, pointing out that these findings are unlikely to have occurred by chance.

The results of our study are concordant with previous research that found that socioeconomically disadvantaged patients report worse experiences with healthcare [17, 21–28]. Our results add to the literature on socioeconomic inequalities in patient experience specific to patients with cancer, which has been limited. Individuals with fewer financial resources reported worse experiences with ‘received information on getting financial help’, ‘support at home from health/social services’ and ‘care from health/social services during treatment’. This might indicate that the current Swiss health system is not responding enough to the needs of socioeconomically disadvantaged people, especially in supporting them with healthcare costs that are not covered under the basic health insurance. Compared to other European countries with universal health insurance coverage, the Swiss health system involves relatively high health insurance premiums and out-of-pocket expenditures. This context may contribute to lower income individuals forgoing healthcare due to cost, which has been evidenced in Switzerland [34, 63–65], as well as to socioeconomic inequalities in healthcare, including cancer screening use [66–69]. These inequalities seem to persist despite the provision of health insurance subsidies to eligible low-income individuals in Switzerland, as previous research has shown that receiving a premium subsidy is associated with forgoing healthcare [34, 63, 65, 66, 70]. It is possible that financially vulnerable individuals and families tend to prioritise expenses other than healthcare, given their economic constraints. In sum, our results suggest that cancer care experiences are affected by the healthcare costs borne by patients in Switzerland. Healthcare professionals and policy-makers should consider this issue since healthcare forgoing and poor patient experience may lead to worse health outcomes in disadvantaged populations.

Our findings are also in line with past research that showed that lower health literacy is associated with inadequate healthcare services use and poor experiences with care [18–20, 48]. Our study significantly adds to this literature since few studies have examined health literacy in relation to comprehensive measures of patient experiences, such as the patient-centred care dimensions [20]. Following the cultural health capital framework [36], several assumptions could be made to explain how health literacy influences patient experience with care in our results. Indeed, patients with lower health literacy may face difficulties in understanding and engaging with medical information and navigating a complex healthcare system. Limited health literacy may hinder patients’ capacity to understand the risks and benefits associated with cancer treatment, and to follow instructions from providers [18, 40, 71, 72]. Moreover, previous studies also showed that lower health literacy can affect the information exchange process during doctor visits, potentially limiting individuals’ ability to engage in effective and meaningful patient-doctor communication [49, 73, 74]. Health practitioners should pay attention to patients with lower health literacy to ensure that they understand the provided information and strive to improve the quality of their communication. This is crucial to guarantee equal opportunities to receive adequate care and support. In clinical settings, various interventions have aimed at improving communication with patients with low health literacy to facilitate their comprehension of health information and instructions [53, 75–77]. These interventions often include strategies such as simplifying written materials, employing different communication formats (e.g. visual aids, illustrated text, spoken animations), and implementing techniques like “teach-back” (i.e. patients rephrasing important information in their own words) to ensure patients’ understanding and enhance face-to-face communication [75, 77]. Other interventions have aimed at improving the education and training of frontline healthcare professionals to improve their ability to address health literacy needs [78, 79].

Notably, in adjusted models, patients’ health literacy was more consistently related to cancer care experience items than economic status. This substantiates the claim that health literacy bears an independent and direct relation to care experiences. Indeed, while material factors such as economic resources are key determinants of disparities in health and healthcare, as put forward by the fundamental cause theory, underlying mechanisms affecting the experience of patients with the healthcare system might be found in immaterial factors, such as cultural resources and abilities. This interpretation is in line with Shim’s “cultural health capital” theory [36], which stresses the role of patients’ cultural competencies, and patient-provider interaction and exchange of information, in producing inequalities in care.

In several studies, patients with higher education levels reported more negative evaluations of healthcare, although results were not always consistent [24, 80, 81]. One may expect that these patients experience better care due to their higher health literacy resources. However, a higher education level may also increase expectations, leading patients to underrate their care experiences. A potential response bias might also stem from the different response patterns of individuals of lower and higher socioeconomic status when answering patient experience questions [80, 82]. Additionally, education level may be a proxy for both health literacy and socioeconomic status, potentially conflating distinct effects and yielding contradictory results. Hence, our study adopted a more direct measurement of health literacy, rather than relying on a proxy such as education level, by simultaneously controlling models for both individuals’ economic status and health literacy, thereby providing a better assessment of these variables and disentangling their effect in our analyses.

The findings of this study carry significant implications for practitioners, policy-makers and researchers, as they show that economic status and health literacy level are associated with patient experiences with care. Given the influence of economic status and health literacy, ‘one-size-fits-all’ approaches in cancer care are likely inadequate for improving patient experience equally. Indeed, the diversity in patient experiences and their distinct characteristics needs to be taken into account. Patient-centred cancer care should not only be tailored to address patient needs and preferences, but also to tackle persistent social and structural inequalities among them. Patients with low health literacy and low income will require more attention and targeted interventions to ensure that care delivery responds to their specific conditions. They may benefit from tailored information and enhanced patient navigation to ensure that care is provided and explained in accessible ways. For example, a study found that the presence of care coordinators strongly improved care coordination, particularly benefiting patients with low health literacy [83]. This suggests that having care coordinators in the healthcare team providing tailored information may facilitate navigation within the healthcare system for disadvantaged patients with low health literacy and improve their experiences with care [84]. Additionally, research showed that clinical nurse specialists played an important role in improving experiences of care coordination, participation in treatment decisions, and feeling treated with respect and dignity [85, 86]. As Nutbeam and Lloyd [53] highlighted, there is a need to shift focus from individual-level interventions to organisational- and community-level interventions, such as efforts aiming at reducing organisational complexity within healthcare systems to improve accessibility and understanding of health information. Known as organisational health literacy (OHL), these initiatives strive to create health-literate environments that facilitate patient navigation and comprehension [78, 87]. However, research evaluating the effectiveness of such interventions in mitigating inequalities in patient experiences has been limited [53].

Furthermore, in light of the increasing prevalence of virtual care modalities like e-health and telemedicine – further accelerated by the global COVID-19 pandemic and its consequent limitations on healthcare access [88] – the significance of factoring in patients’ health literacy levels becomes even more pronounced. Indeed, recent studies have pointed out inequalities in the use of virtual care and electronic patient communication, e.g. email communication [89, 90]. Given the persistence of digital divides, understanding how health literacy is related to the experiences of patients with remote consultations and supports is essential [91, 92]. Research should further examine how such changes in care delivery, including e-health and telemedicine, may have affected inequalities in patient experiences, and how care experiences may be effectively and equally improved in this context.

The following strengths of our study are worth underscoring. First, we conducted a comprehensive analysis of specific patient experiences using a PREM cancer-specific validated questionnaire that encompasses the eight key dimensions of patient-centred care, as well as key aspects of the cancer care pathway – from diagnosis to treatment and follow-up. Second, this is the first study to assess inequalities in cancer patient experiences across multiple hospitals located throughout Switzerland and in a large sample of patients. Only one study has examined such inequalities in four hospitals located in the French-speaking region of Switzerland [93]. Third, our study contributes to the limited literature on socioeconomic and health literacy inequalities in care experiences among patients with cancer by using PREMs rather than satisfaction measures. This is important since research has usually examined inequalities in patient satisfaction (Batbaatar et al., 2017), while satisfaction is only one facet of patient experience, influenced by various factors that may be unrelated to the direct experience of care services.

Our study also has limitations that need to be considered when interpreting the results. This study is based on a cross-sectional survey data, which does not allow for inferring causal relationhips. We could only consider a limited number of control variables measured in the survey, leaving room for potential unmeasured confounding effects. Our findings reflect care experiences as reported by patients who participated in the survey and were cared for in the participating hospitals. Given the focus of our study on hospitals, future research may assess the care experiences of patients in ambulatory/outpatient centres and private practices. Even in a survey with a relatively high response rate, there is always potential for non-response bias. That is, past research showed that survey participants tend to be younger and more advantaged socioeconomically [94, 95] compared to non-responders, which may affect both the survey’s representativeness and the rating of patient experiences. Unfortunately, information on non-responders was not accessible for the SCAPE-2 survey. Time since diagnosis could potentially influence the recall of care experiences in different ways. However, previous research found marginal differences in patterns of care experience when analyses were restricted to patients diagnosed within the past year [96]. Some outcome variables had a smaller sample size due to the exclusion of ‘not applicable’ responses, potentially affecting statistical power and increasing the likelihood of Type II errors by reducing the ability to detect true associations for these outcomes. Additionally, cancer patient surveys inherently focus on survivors, which excludes patients with shorter survival periods [96, 97]. Finally, our study focused on economic status and health literacy, while further patients’ characteristics may influence their experience with cancer care. For example, we did not examine the potential effect of family and social support on patient experience, as these aspects were not included in the SCAPE-2 survey. A recent systematic review stressed the role of patients’ health status, cancer type, prognosis and stage of disease, as well as survey collection methods, in influencing patient-reported experiences [17]. In this review, being from a lower socioeconomic status or an ethnic minority group, or having a poorer mental health status, were also found to relate to worse cancer care experiences. Future research should pay attention to these determinants, and their potential interaction effects on different cancer care experience outcomes.

To conclude, PREMs have been developed both as a quality indicator for healthcare organisations and systems and as a research topic across different care settings, including cancer care. The present study evidenced inequalities in cancer care as patients with less economic and health literacy resources reported worse experiences with cancer care across eight dimensions of patient-centred care. Such insights into the disparity of experiences between more advantaged and disadvantaged patients may support healthcare professionals and policy-makers in prioritising initiatives to enhance cancer patients’ encounters with the healthcare system. Our findings highlight that cancer care efforts are not yet effectively meeting the needs of disadvantaged patients, implying that a ‘one-size-fits-all’ approach to care may not equally enhance patient experience. Thus, care systems may need to be redesigned considering patients’ socioeconomic and health literacy resources, along with the dimensions of patient-centred care and the patients’ care pathway.

## Data Availability

While the dataset generated and analysed in this study is not publicly available, it can be accessed upon reasonable request from the data.unisante.ch repository (DOI:10.16909/DATASET/38).
